# A case of pseudohyperkalemia in a patient presenting with leucocytosis and high potassium level: a Case Report

**DOI:** 10.1186/1757-1626-3-73

**Published:** 2010-02-25

**Authors:** Alice Kim, Benjamin Biteman, Umer F Malik, Shahzad Siddique, Mersadies R Martin, Syed A Ali, Nadeem Maboud, Sabiya Raja, Alison Zachry, Ahmed Mahmoud

**Affiliations:** 1 Department of Emergency Medicine, Cook County Hospital (W Polk St), Chicago, IL (60612-3764), USA; 2 Department of Internal Medicine, San Joaquin General Hospital (West Hospital Road), French Camp, Ca(95231), USA; 3 Saint George's University School of Medicine, Grenada, West Indies; 4 Department of Geriatric Medicine, Mayo Clinic (1st St SW), Rochester, MN 55905, USA; 5 Department of Internal Medicine, Agha Khan Hospital, Karachi, Sindh, 74800, Pakistan; 6 Department of Surgery San Joaquin General Hospital, 500 West Hospital Road French Camp, California, CA 95231, USA

## Abstract

Pseudohyperkalemia can appear in a variety of settings and should be recognized early. Treatment of pseudohyperkalemia can lead to an inappropriate decrease of actual serum potassium levels which may lead to life threatening conditions. In the case presented, an 81-year-old male presented with massive leucocytosis and an extremely elevated potassium level. This case report emphasizes the importance of recognizing pseudohyperkalemia in a patient with a severely increased potassium and WBC level; such patients may be clinically asymptomatic or may have a normal ECG.

## Case Presentation

An 81 year old Caucasian male presented to the emergency department (ED) complaining of blurred vision. One year ago he was diagnosed with Chronic Lymphocytic Leukemia (CLL) via lymph node biopsy and was unfortunately lost to followup. Two days prior to his presentation to the ED he was seen by an ophthalmologist for the same complaint. After a dilated eye examination the physician instructed him to go the ED after discovering a retinal hemorrhage.

In the ED, the WBC measured 796,000 per cubic millimeter and serum potassium was 7.5 mmol/L. Subsequently, he was given intravenous calcium chloride, dextrose 50%, insulin and kaaxylate. A 12 lead ECG did not reveal any changes.

Repeat serum potassium was drawn after the initial treatments revealing an increased measurement from to 9.8 mmol/L. A repeat ECG did not show any new changes and the patient continued to be asymptomatic. Verification to the lab was conducted to rule out hemolysis or clotting.

Calcium chloride, dextrose 50%, and insulin were repeated, and a higher dose of oral Kayexalate was given. The patient was quickly admitted to the telemetry unit for close monitoring. Potassium was again drawn in order to verify that the potassium levels were decreasing, however the serum potassium level continued to increase. Repeat potassium 16 hours after the first potassium values were reported was 11.8 mmol/L.

After extensive research of the literature it was identified that the extremely high leukocytosis was most probably the cause of the pseudohyperkalemia. Our research prompted the use of a more immediate and mechanically benign method utilizing a gas analyzer that returned a potassium level of 2.4 mmol/L.

## Discussion

Hyperkalemia is a life threatening electrolyte derangement that must be quickly recognized and treated. Normal serum potassium levels are between 3.5-5.0 mmol/L. Mild hyperkalemia ranges from 5.1-6.1 mmol/L, moderate between 6.2-7.0 mmol/L and severe above 7.0 mmol/L.

Cardiovascular and neurologic dysfunctions are the primary manifestations of hyperkalemia. The most serious manifestation of hyperkalemia are due hyperexciteablilty of tissues. Patients may have a variety of dysrhythmias, including second- and third-degree heart block, wide-complex tachycardia, ventricular fibrillation, and even asystole. Studies validate a good correlation between the degree of hyperkalemia and ECG changes [1]. The ECG can provide valuable clues to the presence of hyperkalemia of which peaked T waves are the first characteristic manifestation. Further rises are associated with progressive ECG changes, including loss of P waves, and widening and slurring of the QRS complexes. Eventually, the tracing assumes a sine wave appearance, followed by ventricular fibrillation or asystole [2].

Neuromuscular signs and symptoms of hyperkalemia include muscle cramps, weakness, paralysis, paresthesias, tetany, and focal neurologic deficits [2]. Severe hyperkalemia should be promptly identified and treated as it can quickly lead to death. The treatment of hyperkalemia is initiated by membrane stabilization with either calcium gluconate or hypertonic sodium chloride, and then by redistribution of potassium using short acting insulin and dextrose, and/or Albuterol. Furthermore, potassium may be elmininated in the body by use of either loop diuretics or a resin binder such as, Kayexalate. emodialysis should be considered for elevated potassium levels that are refractive to other treatments, if available it is the most reliable and definitive method in decreasing serum potassium levels [3].

As noted in the above case, a diagnosis of pseudohyperkalemia carries with it an absence of ECG changes consistent with the serum potassium measurements. As potassium levels attain 10 mmol/L, SA nodal conduction no longer exists and passive junctional pacemakers emerge producing an accelerated junctional rhythm. Widening of the QRS complex and the T-wave with obliteration of the ST-segment follows. The QRST is replaced by a smooth diphasic sine wave. This finding is a pre-terminal event unless treatment is initiated immediately [4]. The fatal event is either asystole, as there is complete block in ventricular conduction, or ventricular fibrillation.

There are a few hypotheses of the causes of falsely elevated potassium levels in patients with extreme leukocytosis. One theory is from 1975, prior to the routine use heparinized tubes for collection sampling, postulating that potassium is released from the white blood cells during the clotting process [5]. As all of our samples were collected into heparinized tubes and no clotting was noted by the laboratory staff we will reject this theory in our patient. A more recent case report postulated that hyperkalemia may be caused by the combination of the fragility of the leukemic lymphocytes and the mechanical stress the vacuum tubes have on the cells as the samples are collected causing release of intracellular potassium into the serum[6]. Another likely explanation of the pseudohyperkalemia is that severe leukocytosis will have higher consumption of metabolic fuels that may lead to impaired Na/K ATPase pump activity which may contribute to release of potassium from the high number of white cells [7].

Our conventional method of measuring serum potassium level was not correct and in order to minimize the time and mechanical stress of the sample collection we used the Bayer Gas analyzer Assay from an arterial blood gas syringe [7]. The sample collected with this method showed a serum potassium level of 2.4 mmol/L. Hypokalemia was quickly recognized and potassiumlowering therapy was discontinued.

## Conclusion

The exact mechanism of pseudohyperkalemia in extreme leukocytosis is not clear., However, the presence of pseudohyperkalmia should be strongly suspected with elevated potassium, the absence of clinical signs of hyperkalemia, a normal ECG, and the presence of extreme leukocytosis.

## Abbreviations

CLL: Chronic Lymphocytic Leukemia; ED: Emergency department.

## Consent

Written informed consent could not be obtained because the patient was lost to follow-up. Despite repeated attempts we were unable to trace the patient or their family. Every effort has been made to keep the patient's identity anonymous. We would not expect the patient or their family to object to publication.

## Competing interests

The authors declare that they have no competing interests.

## Authors' contributions

AK, UFM* and BB were major contributors in writing and reviewing the manuscript.

SS and SAA were major reviewers of the manuscript.

MRM, NM, SR, AZ were involved in reviewing the manuscript and data gathering.

AM was the attending involved with the case and reviewed the manuscript.

All authors have read and approved the manuscript.
